# Adaptation of an Emotional Stroop Test for Screening of Suicidal Ideation in Portugal

**DOI:** 10.3390/bs12080281

**Published:** 2022-08-12

**Authors:** Graça Esgalhado, Henrique Pereira, Patricia Silva

**Affiliations:** 1 Department of Psychology and Education, Faculty of Social and Human Sciences, University of Beira Interior, Pólo IV, Estrada do Sineiro, s/n, 6200-209 Covilhã, Portugal; 2 Institute of Cognitive Psychology, Human and Social Development (IPCDHS), 3000-115 Coimbra, Portugal; 3 Research Centre in Sports Sciences, Health Sciences and Human Development (CIDESD), 5001-801 Vila Real, Portugal

**Keywords:** emotional Stroop test, screening, suicidal ideation, Portugal

## Abstract

Cognitive instruments, especially those with emotional components, may be useful to address the limitations of self-report scales commonly used to assess suicidal ideation. The aim of this study was to develop an emotional Stroop test for screening suicidal ideation in Portugal. The project was developed in five phases using different samples for each phase. The first two phases were focused on the formulation of the potential words that would compose the slides. For this purpose, five biology teachers (neutral slide) and five mental health professionals (positive and negative slides) were invited to help choose the words that were most representative for each slide. The third phase validated the words defined in the previous phase. In this phase, 300 university students participated (*M*_*age*_ = 21.66; SD = 3.67; 68% female). They rated the words on a Likert scale in terms of their frequency of use, familiarity, level of understanding, and degree of image evocation. In the fourth phase, the researchers developed the complete version of the test, which consists of three slides with neutral, positive, and negative emotional stimuli, consecutively. Finally, in the fifth phase, we validated the final version of the test through a comparative study between a clinical group and a non-clinical group, each one composed by 50 participants (*M*_*age*_ = 32; SD = 9.70; 55% female). Results indicated that the clinical group demonstrated significantly higher scores for depression and suicidal ideation and lower scores for the three Stroop tasks. Words related to negative emotions were strongly correlated with suicidal ideation. Finally, the three Stroop slides explained 74.1% of the variance in suicidal ideation. These findings suggest that this test can be a viable complementary measure in the psychological assessment of suicide ideation, and intervention in the field of suicide prevention in Portugal.

## 1. Introduction

Suicide is a complex phenomenon which is classified by the World Health Organization as a serious public health concern, since 800,000 people die due to suicide every year [1]. The occurrence of suicide involves multidimensional determinants, such as biological and genetic, psychological, psychiatric, social, and environmental factors [2], with suicidal ideation being one of the main predictors of suicide attempt [3,4]. The relationship between psychological factors and suicidal ideation is complex but involves perceived burdensomeness and internal entrapment, as well as feelings of defeat, external entrapment, and thwarted belonginess [5]. Suicidal ideation is a relatively recurrent and stressful experience, given that about 10% of people have had some type of suicidal thoughts throughout the life cycle [6]. However, anticipating this type of event is not easy, as the psychological assessment of suicidal ideation involves cognitive, emotional, and behavioral measures before the occurrence of suicidal thoughts [7]. Factors such as gender (women have greater suicidal ideation than men) [8], rumination and cognitive inflexibility [9], and previous suicidal ideation and attempt experiences [10] seem to be important predictors of the occurrence of suicidal ideation.

Suicidal ideation refers to a vast set of cognitions, emotions, and behaviors associated with the thought of ending life, often associated with ideas, intentions, motivations, and plans to carry out suicide [11]. Since the assessment of suicidal ideation depends on self-report, several difficulties arise in the operationalization of this assessment, as people tend not to manifest their intentions [12] and tend to deliberately deny or hide their thoughts to avoid interventions or hospitalizations [13]. This reinforces the importance of developing viable alternatives to assess suicidal ideation, namely through the assessment of cognitive and emotional functioning.

The Stroop task [14] is a well-known cognitive task to assess the attentional processing of simultaneous information in the context of selective attention, cognitive set shifting, and inhibition of responses [15,16]. In the original version of the color-word Stroop task (CW-Stroop) [17], three slides are presented. The first slide contains words of the colors “blue”, “green”, “red”, and “yellow” printed in black. The second slide contains rectangles printed in blue, green, red, or yellow (“color-naming” condition). The third slide shows the same words as the first slide printed in an incongruous color (for example, the word “green” printed in blue). Hence, Stroop interference allows the assessment of attentional capacity and bias.

The Emotional Stroop Test introduces changes to the CW-Stroop by replacing colored words with neutral and emotional stimuli (for example, the word “death” printed in blue compared to a neutral word of the same color) [18]. In individuals without psychopathology, the Emotional Stroop Test does not cause attentional bias. However, in individuals with psychopathology, it is common to observe some kind of attentional bias, interpreted as threatening [19]. Several studies have analyzed Stroop interference in patients with anxiety and depression disorders [20,21], post-traumatic stress [22], and suicidal behavior [8,23].

Therefore, this work aims to develop methodological procedures in order to contribute to the evaluation of the Emotional Stroop Test for the detection of suicide ideation as a measure of suicidal risk. Unlike most previous studies that focus on clinical populations [24,25], this study focuses on associating the Stroop task to assess differences in attentional functioning when compared to specific levels of suicide ideation in clinical and non-clinical populations in Portugal. We propose that individuals that report higher levels of suicide ideation will present more difficulties in attentional control, hence being at a higher risk of suicide, because they are unable to inhibit negative thoughts and direct attention toward task-relevant information. In contrast, participants with lower levels of suicide ideation will be able to control attentional functioning and are thus less likely to focus on negative thoughts. Hence, we predict that participants with more suicide ideation will show more difficulties inhibiting negative words. 

## 2. Materials and Methods

This study took place in four phases: (1) obtaining the words for the neutral slide, (2) obtaining the words for the positive and negative slides, (3) word validation, (4) formulation of the Emotional Stroop Test, and (5) validation of the Emotional Stroop Test by comparing a clinical and a non-clinical group. For each phase, different samples were used. We explain ahead each phase with greater detail.

### 2.1. Phase 1—Words for the Neutral Slide

To carry out the first phase of the investigation, we used five Biology teachers from a school in the municipality of Covilhã, Portugal to become formal judges. All teachers had a university degree in biology as a minimum educational qualification. As this task had a similar structure to the classic Stroop test, the neutral slide consisted of non-activating words. We chose words belonging to the “fruits” category. For the selection of these words, the Portuguese lexicon was used, and 48 words were withdrawn from the search. An authorization was requested to apply the questionnaire to the school principal in order to allow the participation of selected judges who were present with the words and were asked to classify them as belonging or not to the fruit category. The list of the 48 words belonging to the fruit category is presented in Table 1.

Of these 48 words, 10 were selected to be included in the slide. The choice criterion was due to the fact that the ten words were classified by all the judges as belonging to the fruit category and taking into account the four colors to be used in the Emotional Stroop—yellow, red, blue, and green—the chosen words have the same number of syllables (in Portuguese) of the color in which they were be printed (highlighted in Table 1).

### 2.2. Phase 2—Words for the Positive and Negative Slides

To carry out the second phase of the investigation, an intentional sample was used, consisting of five specialists in the field of mental health. Of these five specialists, three were clinical psychologists, one was a psychiatrist, and last one was child psychiatrist. For the construction of the positive slide and negative slides—a bibliographic research was developed on words associated with the issue in question (suicide ideation) and the stage of the life cycle in which the participant was at. Seventy-four words were presented to the specialists’ panel, who were then asked to classify the words on a Likert-type scale with values ranging from −2 (very negative words) to +2 (very positive words). Other words that the judges considered pertinent could be added to the initial list.

Of the initial 74 words, only 20 were selected. Ten were classified as positive and ten were classified as negative. Each ten words were chosen in accordance with an obtained common score by the five judges of above 60%—i.e., the words were validated by the five judges when their agreement was common for at least three of them. In addition to this criterion, and similar to the neutral blade, the number of syllables that the words have in Portuguese was also taken into account, such that the chosen words had the same number of syllables corresponding to the number of syllables as the color of the words in the which they were printed (Table 2).

### 2.3. Phase 3—Word Validation

In this phase we used a convenience sample of 300 university students who attended the University of Beira Interior in Portugal. The mean age was 21.66 (SD = 3.67), 68% were female, and 32% were male. Of this 300, 5% studied architecture, 14.7% sociology, 23.3% sports Science, 30.7% psychology, 8.7% marketing and 17.7% medicine.

After obtaining the three sets of words, a protocol consisting of five pages was elaborated. The first page contained a brief explanation of the study, as well as instructions. The second page contained sociodemographic information. The remaining pages presented a brief explanation of the task that was being asked, and three tables with words in which the participants should comment. The protocol application took place in a classroom of the university facilities after informed consent was obtained. Anonymity and confidentiality were guaranteed. This sample assessed the three sets of words stimuli—neutral, negative, and positive—on a Likert-type scale, ranging from 1 (nothing) to 4 (very) in relation to the “Frequency of Use”, (if they were used in everyday life) to “Familiarity” (if they were more or less familiar), to “Level of Understanding” (if their meaning was understood), and to the “Degree of Image Evocation” (if it was easy or difficult to mental visualize the image corresponding to the word). A total of 51% of participants considered all the words frequent, familiar, understandable, and evocable. 

### 2.4. Phase 4—Formulation of the Emotional Stroop Test for Screening of Suicide Ideation

In this phase, we started to create the Stroop test for screening of suicide ideation. Like all Stroop procedures, whether classic or emotional, each slide was printed in A4 format (21 × 30 cm). Each slide contained 100 elements distributed over five columns of 20 elements. All words were randomly distributed, and the same word was not allowed to appear twice in a row in the same column, just as the same color was not allowed to appear twice in a row. The words that made up the three slides of the Emotional Stroop Test for suicide ideation are presented in Table 3.

**Figure 1:**
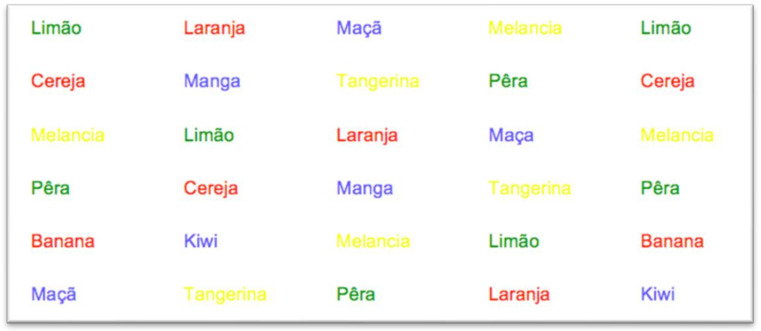
The ten neutral words.

**Figure 2:**
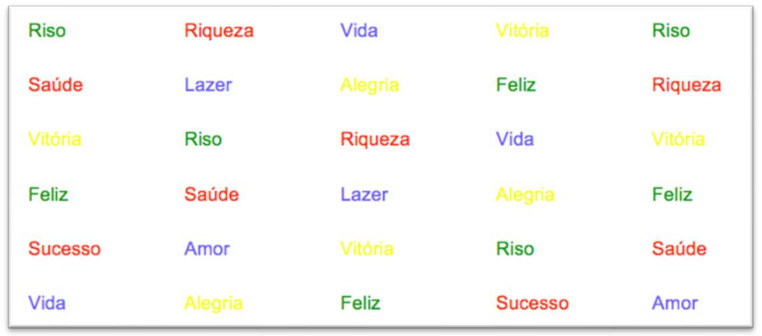
The ten positive words.

**Figure 3:**
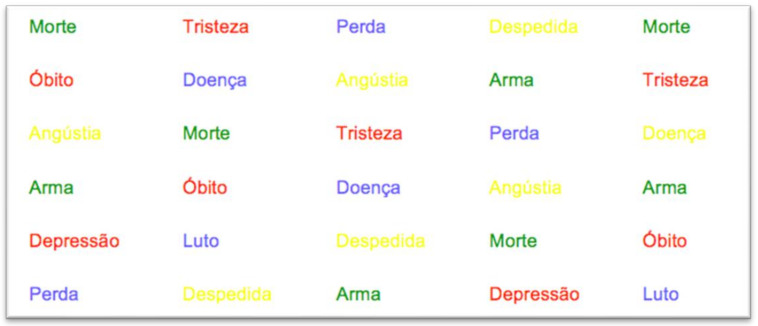
The ten negative words.

Figure 1, Figure 2 and Figure 3 are excerpts from the Emotional Stroop Test for screening of suicide ideation in Portuguese. The first slide printed the ten neutral words in colored ink—blue, yellow, green, and red (Figure 1).

The second slide contained the words that were classified as positive, also printed in color. Figure 2 shows an excerpt of that slide.

The third slide contained the words that were classified as negative, also printed in color. Figure 3 shows an excerpt of that slide.

### 2.5. Phase 5—The Final Study: The Emotional Stroop Test for Screening of Suicide Ideation and The Suicide Ideation Questionnaire

In this final step, we used 100 participants divided into two groups: clinical (*n* = 50) and non-clinical (*n* = 50). In the clinical group, the presence of depressive symptoms was defined as an inclusion criterion, assessed through the Beck Depression Inventory [26], and the exclusion criterion was the presence of other diagnosis. In the non-clinical group, the presence of a psychopathological symptoms was used as an exclusion criterion. Participants were aged between 20 and 59 years, with an average age of 32 years (SD = 9.70). With regard to gender distribution, 55 subjects (55%) were female and 45 (45%) were male. Regarding marital status, 67% of participants were single, 30% married, and 3% divorced. A total of 27% of participants were students, 16% were unemployed, 8% were nurses, 6% were operational assistants, 5% were engineers and managers, 4% were social workers, and teachers, 3% were sociologists, 2% were psychologists, and 1% were entrepreneurs. Regarding education, 48% had a university degree, 35% had secondary diploma, and 7% had basic school diploma. Homogeneity tests were used to test similarity between the clinical and non-clinical groups for sociodemographic characteristics. The only main difference was having or not having clinical symptomatology for depression. 

A sociodemographic questionnaire was used to characterize the participants on age, gender, professional status, education, and a self-assessment item to detect the presence of psychic comorbidities.

The prevalence of suicidal thoughts and cognitions was assessed using the Suicidal Ideation Questionnaire (SIQ). This instrument is a Portuguese version of the Suicide Ideation Questionnaire, originally created by Davis [27] and translated and adapted to Portuguese [28]. The SIQ allows analyzing the severity of suicidal thoughts in adolescents and adults, and assesses a hierarchy of thoughts related to suicide, which oscillate between little and very serious. It consists of 30 items on a Likert-type scale, ranging from “I never thought” to “I always thought”, in a growing trend of severity. For evaluation purposes, each item is rated from 0 to 6, respectively. The total scores can range from 0 to 180. In the Portuguese version, a score of 23.04 was defined as the average for the population (SD = 25.65). A Cronbach’s alpha coefficient of 0.96 was obtained in the present study, indicating excellent reliability for the SIQ.

The clinical sample was collected from the psychiatric department of a local general hospital. After obtaining a formal consent to access to patients from the ethics committee, the sociodemographic questionnaire, the Emotional Stroop Test created in this study, the Beck Depression Inventory, and the Suicidal Ideation Questionnaire were used to collect data. All ethical principles of anonymity and confidentiality were guaranteed. The non-clinical sample was collected via formal and informal community contacts using the snowball technique to collect participants’ responses to the same measures.

Immediately before applying the set of instruments, a quick detection of any deficiencies in color perception (for example, achromatism, or dichromatism) was performed by presenting color squares (yellow, green, blue, and red) and asking participants to identify the respective colors. Subsequently, some indications and instructions were given in order to ensure the understanding and proper performance of the Emotional Stroop Task by naming the color in which the word was printed aloud as quickly as possible, while ignoring its meaning, in 45 s. The BDI and the SIQ were also applied. Throughout the sample collection procedure, a face-to-face approach was used. Participants needed, on average, 10 to 20 min to answer the questionnaires and requested little support in clarifying any items.

### 2.6. Data Analyses

Quantitative results were analyzed using IBM SPPS Statistics, Version 27.0. After data cleaning, descriptive statistics, including frequencies, means, and standard deviations were established. In order to compare differences between samples, t-student tests were conducted. Finally, correlational and linear regression analyses were also performed.

## 3. Results

A normal distribution was found for all variables under study after conducting the Kolmogorov–Smirnov test. Regarding depression symptoms, participants from the non-clinical group scored significantly lower than participants from the clinical group, *M_non-clinical_* = 3.06, SD = 2.54, and *M_clinical_* = 43.88, SD = 11.97, respectively [t(98) = −23.57; *p* < 0.001)]. Regarding suicide ideation, participants from the non-clinical group scored significantly lower than participants from the clinical group, *M_non-clinical_* = 2.22, SD = 3.12, and *M_clinical_* = 83.46, SD = 19.97, respectively [t(98) = −28.41; *p* < 0.001)] (Table 4).

For the Emotional Stroop Test for screening of suicide ideation, a comparative analysis between non-clinical and clinical groups was performed for all three types of slides with neutral, positive, and negative words, assessing the number of correct words evoked. Results shown in Table 5 indicate that significant differences were obtained for all three conditions (*p* < 0.001). Non-clinical participants scored higher for all three conditions.

A correlational analysis between the number of negative words on slide three (negative words) and levels of suicide ideation. A very strong, negative, and significant association was found (r = −0.840; *p* < 0.001). Table 6 describes these correlations.

Finally, simple logistic regression was performed to assess the predictive value of neutral, positive, and negative words in suicide ideation. As shown in Table 7, the model was found to be significant, and indicates that the Stroop slides explain 74.1% of the variance in suicide ideation levels, with greater contribution from the negative words (slide 3).

## 4. Discussion

The present study sought to develop an Emotional Stroop Test as a suicidal risk measure for the Portuguese population. The test aims to assess differences in attentional functioning when compared to specific levels of suicide ideation. We hypothesized that people with higher levels of suicidal ideation performed worse on the Stroop task. There were then four phases of test formulation in Portuguese and one phase for the validation of the test through the application of the final questionnaire to a clinical sample (*n* = 50) compared to a non-clinical sample (*n* = 50). The clinical sample had higher scores for depression and suicidal ideation and lower scores for correct answers on the 3 slides of the Stroop task. Suicidal ideation was strongly correlated with negative words, which in turn contributed to a greater explanation of the variance in levels of suicidal ideation. All results were significant.

Our findings corroborate previous studies that demonstrated that the Emotional Stroop Test is an important tool for the assessment of suicidal ideation, as it fills gaps in commonly used self-report scales [10,29,30]. In fact, this test will be beneficial for the field of psychological assessment in Portugal, as it considers specific criteria and characteristics related to suicidal ideation that other measures are not prepared to assess. This is due to the fact that this test can measure how well a person can use their ability to control their automatic reactions to words or images, which is called the “Stroop effect” [14,18]. From the use of words with positive, negative, and neutral emotional stimuli, it is possible to assess attention bias, response inhibition, and cognitive flexibility—variables that are related to a person’s emotions and affective states and have already been widely related to suicidal behavior [31,32,33].

In fact, the Stroop paradigm has been used to demonstrate attentional bias in different clinical populations. Studies with these populations have shown that participants have longer reaction times when exposed to stimuli associated with their dysfunctional behavior; that is, they tend to name the color of words related to their problems, or emotionally relevant stimuli, more slowly than those with neutral emotional valence. It is also verified in different investigations with the Emotional Stroop Test, that the scores obtained in the three slides by the control group are higher than those of the clinical group. We can infer that participants in the clinical group may have a psychological impairment that influences their performance, possibly with implications for a decrease in the speed of response processing, which explains why they also score less on the neutral slide than participants in the control group. However, it is in the negative slide, as would be expected, that these differences are accentuated, which corroborates the results of different versions of the Emotional Stroop Test that use the scores in the negative slide as an indicator of the interference of the meaning of the words in the execution of the requested task, the naming of the color in which they are written.

There are also operational aspects that support our tool as a useful measure for the assessment of suicidal ideation. The main one is related to the application time. This test can be used as a quick and easy way to detect suicidal tendencies (participants needed, on average, 10 to 20 min to complete the entire protocol). In addition, this type of test is especially effective for assessing these tendencies in people who would not normally present/respond to a suicide assessment [34,35], or for those who might have response biases on the self-report scales, such as people of clinical populations [10,36,37]. Hence, this is a useful and practical test in clinical settings since its application is easy and quick, providing immediate screening clues for suicidal ideation. In addition, our protocol can be administered in a variety of settings, including psychiatric hospitals, private practices, and schools, which increases the tool’s reach in suicide screening. 

The information obtained through this Emotional Stroop Test can be used by mental health professionals to assist in the assessment and monitoring of a person’s or group’s suicide risk, which would allow them to direct people with suicidal ideation to appropriate services and determine which prevention measures need to be taken. In fact, different types of treatment can be more or less effective depending on whether or not the person is conscious of the suicidal ideation and what the level of suicidal ideation is [38], which can be verified through the Emotional Stroop Test. Psychology researchers can also benefit from this tool to study suicidal ideation in different populations. With a neuropsychological dimension, this test can be useful to complement analyzes with regard to assessments, comparisons and discovery of patterns and potential risk groups. This is also the case when a differential diagnosis between impulsive behaviors associated with attention deficit with hyperactivity disorder (ADHD) and impulsive behaviors associated with depression, because the Emotional Stroop Test is sensitive to attentional biases caused by negative words related to each disorder. 

In addition, we need to understand our test from a public health perspective. In Portugal, around 3 people die every day from intentional self-harm (suicide) [39]. In 2017, suicide represented a loss of about 15 potential years of life [39]. In addition, the country is the first in the European Union with more people to suffer from chronic depression, which has a direct connection with suicidal ideation [40]. According to the Lusa Agency and the National Authority for Medicines and Health Products (Infarmed), the sale of antidepressants has also increased considerably in recent years, reaching an increase of almost 100% between 2010 and 2019 [41]. For these reasons, the prevention of suicidal behavior through population screening and the implementation of intervention strategies should be identified as a public health priority [1], which demonstrates the relevance of this study in the context of public health in the country. 

However, despite being an innovative work and with important contributions, this study has its limitations. The first one is related to the validation of the protocol for specific populations. Although our sample had a heterogeneous distribution, the final study was carried out with a diverse audience (different ages, academic degrees, socioeconomic status, etc.), which may have influenced our results. Future studies could benefit from the validation of this test for specific populations (adolescents, elderly people, people from minority groups, among others) and for larger and more representative samples. Validation studies are important to determine whether a psychological test can be used safely, which will contribute to the construction of knowledge on this test in Portugal.

Another limitation is related to the need to understand the colors to perform the test. In this case, those who have difficulties in identifying colors are excluded. This also applies to people with language difficulties or diminished cognitive abilities, who should not be evaluated using this test. For these people, we recommend the use of other assessment measures with a neuropsychological basis and others, which are able to complement the suicide risk assessment with more precision [37]. Finally, this test was based on computer slides and considers the importance of innovation present also in the area of psychological assessment. Future studies could verify the feasibility and relevance of using a mobile application version of this test, which would facilitate its application by professionals who do not have access to a computer at the time of the assessment [42,43].

## 5. Conclusions

We believe that assessing a person’s attentional functioning is fundamental for suicide screening, given that individuals with suicidal ideations have greater difficulties in inhibiting their impulses than healthy individuals. In this sense, this study sought to present the development and feasibility of an Emotional Stroop Test for screening for suicidal ideation in a clinical and non-clinical sample of the Portuguese population. We describe several advantages of our study, as well as important implications for academic, clinical, and public health practice in the country. We also emphasize the importance of identifying suicidal ideation before any suicide attempt, in order to contribute to the promotion of effective actions for each case. Finally, we discuss the main limitations and future directions for research with this tool. We conclude that this Emotional Stroop Test will be beneficial for the area of psychological assessment in Portugal with a focus on cognitive functions, since the test measures selective attention, executive functions, cognitive flexibility, inhibition, and the attention biases than can appears in case of psychopathology. This test can be included in neuropsychological assessment tools that do not depend on self-harm self-report questionnaires and be an important resource to assess suicidal ideation and assist in suicide prevention in people of Portuguese language.

## Figures and Tables

**Table 1:** Neutral words presented to the specialists’ panel.

Fruits			
Fig	Blackberry	Persimmon	Medlar
**Cherry**	Blueberry	Raspberry	**Apple**
Pomegranate	Currant	Nectarine	**Pear**
Date	Strawberry	Pinion	**Banana**
**Grape**	Olive	Peanut	Melon
Lime	Guava	Pistachio	**Watermelon**
**Lemon**	Lychee	Cashew	Sloe
Cider	Pineapple	Raisins	**Orange**
**Tangerine**	Walnut Mango	Plum	Quince
Grapefruit	Papaya	Apricot	Peach
Avocado	Coconut	Chestnut	Almond
Passion Fruit	**Kiwi**	Hazelnut	Arbutus

Note: Words belonging to the Fruit category, to integrate the neutral slide are highlighted in black.

**Table 2:** Positive and negative words presented to the specialists’ panel.

Words					
Cry	**Life**	**Depression**	Qualities	**Loss**	**Success**
Sun	Burden	Help	**Joy**	Change	**Victory**
**Wealth**	Try	Ache	Conflict	Alcohol	Testament
**Death**	**Mourning**	Charm	**Anguish**	Beauty	Survive
Adolescence	**Wealth**	Unemployment	Affable	Development	Rejection
Crisis	**Love**	Loneliness	Aggressiveness	Praise	Parasuicide
**Farewell**	Appeal	Salvation	Employment	Friendship	Anxiety
Drugs	**Passing**	**Laughter**	Sexuality	**Sadness**	Confusion
Color	**Health**	Hope	End	**Recreation**	Identity
**Weapon**	Separation	**Disease**	Humor	Self-harm	Defects
**Happiness**	Transformation	Hanging	Growth	Sacrifice	Youth
Isolation	Pills	Struggle	Impulsivity	Communication	Hopelessness
				Suffering	Suicide

Note: Words belonging to the positive and negative categories to integrate the correspondent slides, are highlighted in black.

**Table 3:** Neutral, positive, and negative words.

Neutral Blade	Positive Blade	Negative Blade
Tangerine	Love	Death
Banana	Joy	Farewell
Kiwi	Victory	Mourning
Watermelon	Health	Depression
Orange	Life	Passing
Apple	Laughter	Loss
Grape	Success	Sadness
Cherry	Wealth	Weapon
Lemon	Happy	Disease
Pear	Leisure	Anguish

**Table 4:** Depression and suicide ideation scores by non-clinical and clinical groups.

Variable	Group	n	*M* (SD)	t (df)	*p*	*Cohen’s d*
Depression	Non-Clinical	50	3.06 (2.54)	−23.57 (98)	0.000 *	81.240
	Clinical	50	43.88 (11.97)			
Suicide Ideation	Non-Clinical	50	2.22 (3.12)	−28.41 (98)	0.000 *	40.820
	Clinical	50	83.46 (19.97)			

* *p* < 0.001.

**Table 5:** Results for neutral, positive, and negative words scores by non-clinical and clinical groups.

Variable	Group	*n*	*M* (SD)	t (df)	*p*	*Cohen’s d*
Neutral words	Non-Clinical	50	60.64 (9.41)	5.92 (98)	0.000 *	−9.280
	Clinical	50	51.56 (5.84)			
Positive words	Non-Clinical	50	64.34 (7.53)	−11.61 (98)	0.000 *	−15.740
	Clinical	50	48.60 (5.93)			
Negative words	Non-Clinical	50	66.32 (7.59)	14.64 (98)	0.000 *	−20.460
	Clinical	50	45.86 (6.32)			

* *p* < 0.001.

**Table 6:** Correlation Matrix.

	1	2	3	4
1. Suicide Ideation	-			
2. Slide 1 (Neutral)	−0.558 **	-		
3. Slide 2 (Positive)	−0.773 **	0.844 **	-	
4. Slide 3 (Negative)	−0.840 **	0.801 **	0.952 **	-

** *p* < 0.001.

**Table 7:** Simple linear regression analysis predicting the suicide ideation.

Variable	*B*	*SE B*	*β*
Slide 1 (Neutral)	1.515	0.461	0.318 *
Slide 2 (Positive)	0.013	0.790	0.003
Slide 3 (Negative)	−3.824	0.593	−1.098 **
*R^2^*	0.741		
*F*	91.728 *		

* *p* < 0.05; ** *p* < 0.001.

## Data Availability

The data presented in this study are available upon request.
